# Agriculture Waste Biomass Repurposed into Natural Fibers: A Circular Bioeconomy Perspective

**DOI:** 10.3390/bioengineering9070296

**Published:** 2022-07-01

**Authors:** Kuzhandaivel Jayaprakash, Atieh Osama, Rajinikanth Rajagopal, Bernard Goyette, Obulisamy Parthiba Karthikeyan

**Affiliations:** 1 Department of Biotechnology, Sri Vinayaga College of Arts and Science, Ulundurpet 606107, Tamil Nadu, India; phycojai@gmail.com; 2 Department of Engineering Technology, College of Technology, University of Houston, Houston, TX 77004, USA; osamaatieh1999@gmail.com; 3 Sherbrooke Research and Development Center, Agriculture and Agri-Food Canada, Sherbrooke, QC J1M 0C8, Canada; bernard.goyette@agr.gc.ca; 4 Institute of Bioresource and Agriculture, Hong Kong Baptist University, Kowloon Tong, Hong Kong; 5 Department of Civil and Environmental Engineering, South Dakota School of Mines and Technology, Rapid City, SD 57701, USA

**Keywords:** natural fibers, primary and secondary sources, fiber processing, banana fibers

## Abstract

Fibers come from natural and fossil resources and are an essential commodity widely used by textile industries. Considering current supply and future demands, the repurposing of agricultural residues into fibers is an eco-friendly, attractive option that might mitigate environmental pollution. In this review, we have summarized multiple alternate secondary sources for fiber production, with a case study using banana plant residual biomass, a common agricultural waste in many developing countries. Specifically, in this review we have compared the different processing methods, e.g., chemical, mechanical, or biological methods, for repurposing agricultural residual biomass (including banana waste) into fibers. The development and analysis of an integrated biorefinery approach is needed to promote the fiber production from various agro-residual biomasses within the framework of circular bioeconomic concepts.

## 1. Introduction

Globally, ~140 gigatons per year (Gt.Y^−1^) of biomass are discarded as waste, contributing to a significant environmental burden (e.g., greenhouse gas emission, soil-water quality deterioration, etc.). This also includes forest biomass (~4.6 Gt.Y^−1^), in which 60% goes for energy generation and the remaining is waste biomass [1]. With an increasing population and the rise of food/fuel debates, there is a great deal of pressure put on the agricultural sectors that are expected to generate more biomass. More demand for and supply of agriculture products has resulted in more disposal of biomass residues and other application of biomass in different fields, such as absorbents, fillers, different ceramic synthesis, etc. The current disposal/utilization pattern of discarded biomasses has shifted from fertilizer production, animal feeding, or burning to biogas/biofuel production and other value-added bio products [2]. Under the concepts of biorefining and a circular bioeconomy, bioenergy/biofuel production using residual biomass has been widely established. However, it has overlooked the potential of recycling options to produce natural fibers due to the techno-economics of process production [3]. Fibers are long, continuous short or long filament polymers that are grouped into the following categories: cellulose (e.g., plant-based biomass), protein (e.g., animal wool or silks), mineral (e.g., asbestos), and synthetic or man-made (e.g., polyesters, fossil-derived nylon) fibers, as shown in Table 1. It is projected that about 134.5 million tons of fibers per year will be required by 2025 (~30% natural and 70% synthetic), of which > 70% of fibers will be going towards textile production [4]. There is some mismatch in the statistics from literature data, since the processed cellulose is also categorized as synthetic/man-made fiber [3]. Asia contributes 95% of the global fiber production (both natural and man-made fibers), while China alone contributes up to 65%. Considering the anticipated demands for the fiber, converting the potential agricultural biomass into fibers reduces pressure on synthetic polymer production using fossil resources. This can be achievable by developing integrated biorefinery and bio processing approaches that produce multiple bio-products. In this review, we have provided the perspective of recycling plant biomass to produce fibers, with a case study to promote circular bioeconomy concepts. Under circular bioeconomy concepts, it is possible to repurpose the fibers many times over to reduce the carbon and energy footprints. Therefore, the purpose of this perspective article is to highlight the possible use of biomass for fiber production and to explain the integrated bio-refinery concepts using banana biomass as a case study.

### 1.1. Natural Fibers and Composition

**Figure 1:**
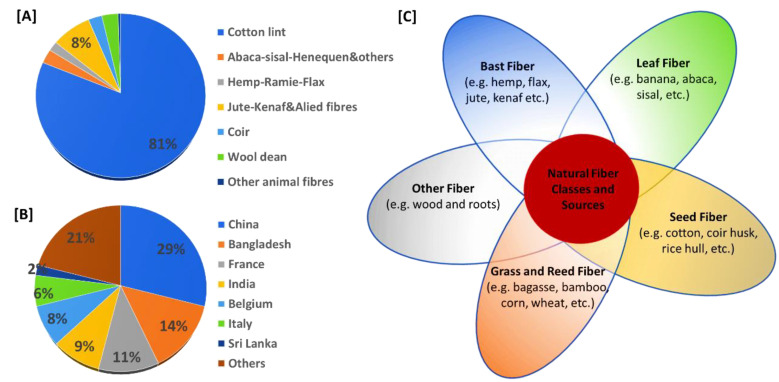
Types of natural fibers, their classifications, sources, and supply. (**A**) Distribution percentage of different types of natural fibers used as of 2018; (**B**) natural fiber supplies; and (**C**) classes of natural fibers and their sources (adapted and modified from [8,9]).

Natural fibers are biodegradable polymers that have zero modification. They are continuous long chain or short chain filaments derived from plants, animals, insects, and minerals [7]. The global production of natural fibers had reached~32 million tons by 2018 (Figure 1A). China dominates plant fiber productions followed by Bangladesh (Figure 1B). Cotton is one of the best examples of a cash crop that is being widely planted (in over 80 countries) to produce natural fibers, i.e., 80% of natural fibers (~26.05 million metric tons by 2018) come from cotton. Cotton consists of cellulosic short filamentous polymers. Figure 1C compiles and groups the diverse classes of primers under primary and secondary fibers based on their sources. The primary fiber producers are solely planted for fiber production (e.g., cotton, flax, bamboo, sisal, hard/softwoods, etc.). Whereas the secondary fiber producers fibers as a byproducts of other primary utilization sources (e.g., banana, coir, rice, bagasse, pandan, pineapple leaves etc.). Banana fiber is also a major substitute for the pulp industries. With so many beneficial properties, the fiber is gaining popularity in the fashion world as one of the best resources for fiber production, with superior properties compared to other synthetic fibers.

In general, natural fibers from plants are relatively low in density and their tensile properties are comparable to those of glass or carbon fibers. Table 2 shows the physiochemical and mechanical properties of different plant fibers. Based on their tensile properties, they are ordered as bast, leaf, seed, grass, reed, hard, jute, cotton, flax, hemp fiber etc., [10]. Cotton fibers are unicellular cellulosic fibers which are known for their beneficial properties (e.g., resistant to alkali, non-irritating, non-contaminating, etc.), low-cost, and applicability in textile productions. Kapok fibers possess superior properties, i.e., high water retention, and are commonly used as insulating materials and for making composite. It is reported that more than 2000 fibers are used to make composite reinforcement materials, with wide applications in medicine, food, civil engineering, cosmetics, and environmental uses [11]. Compared to all other leaf fibers, henequen fibers have a relatively high tenacity, which makes them more suitable for polymer reinforcements [12]. Bast fibers have been used for more than 8000 years in textile production, and they are usually placed in bundles or produced as aggregates, which requires processing to remove any lignin/pectin during extraction.

### 1.2. Fiber Extraction

**Figure 2:**
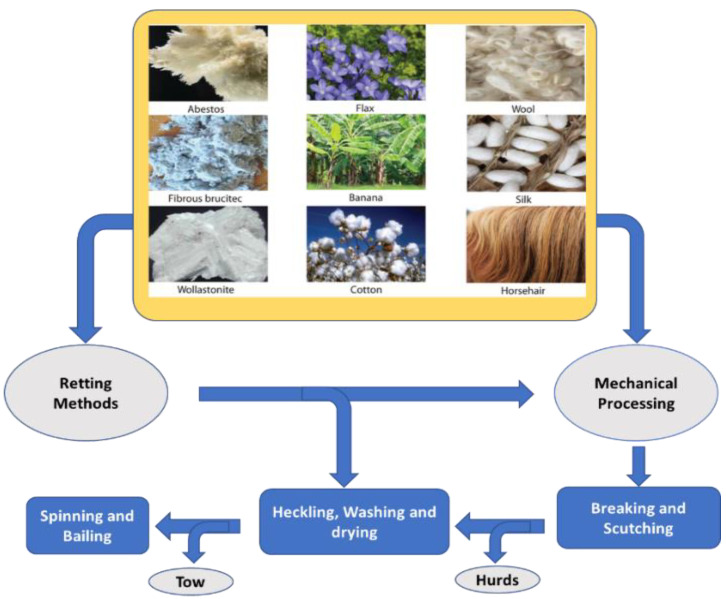
Extraction of bast fibers from source materials.

All fiber is processed by the same method, i.e., retting, or mechanical processing. In some cases, a combination of retting, followed by mechanical extraction, is also used (Figure 2). Retting (or degumming) is a controlled process for removing pectinaceous (gummy carbohydrate materials) substances that hold the fibers as bundles [13,14]. Based on the type of process, retting is categorized as follows:

**Table 2:** Physical, chemical, and mechanical properties of natural fibers [3,15,16,17].

Fiber Class	Types of Fiber	Volume (Tonnes)	Dimension			Chemical Composition			Physical and Mechanical Properties			
			Fiber Length (mm)	Width Fiber (μm)	Fibril Angle (Degrees)	Cellulose (%)	Hemicellulose (%)	Lignin (%)	Density (g m^−2^)	Tensiles Trength (MPa)	Young’s Modulus (GPa)	Elongation at Break (%)
**Bast**	Flax	190,000	0.20–1.40	0.04–0.62	6–10	69.22–71.65	18.31–18.69	3.05–2.56	1.25–1.55	500–900	50–70	2.70–3.6
	Jute	2,700,000	3.00–3.50	60.00–110	7–9	69.21–72.35	12.55–13.65	12.67–13.21	1.3–1.45	300–700	20–50	1.69–1.83
	Kenaf	230,000	0.66–0.82	17.70–26.70	5–10	37.50–63.00	15.10–21.40	18.00–24.30	0.15–0.55	295–955	23.1–27.1	1.56–1.78
**Leaf**	Sisal	247,000	0.85–1.00	100–300	10–25	43.85–56.63	21.12–24.53	7.21–9.20	1.45–1.5	300–500	10–30	4.10–4.3
	Abaca	78,000	2.00–4.00	150–260	6–7	69.23–70.64	21.22–21.97	5.15–5.87	1.42–1.65	879–980	38–45	9–11
	Pineapple	-	3.00–9.00	20.00–80.00	10–15	70.55–82.31	18.73–21.90	5.35–12.33	1.25–1.60	166–175	5.51–6.76	2.78–3.34
	Banana	1,500,000	0.90–4.00	80.00–20.69	9–13	60.25–65.21	48.20–59.2	5.55–10.35	0.65–1.36	51.6–55.2	3.00–3.78	1.21–3.55
**Seed**	Oil plant	-	0.33–50.31	8.30–220.50	40–46	44.20–49.60	18.30–33.54	17.30–26.51	0.7–1.55	227.5–278.4	2.7–3.2	2.13–5.00
	Coconut coir	340,000	0.3–1.00	92.00–314.00	39–49	36.62–43.21	0.15–0.25	41.23–45.33	0.67–1.15	173.5–175.0	4.0–6.0	27.21–32.32
	Kapok	-	2.00–3.00	14.1–18.9	7.3–8.7	65.63–69.87	6.66–10.49	5.46–5.63	0.65–1.47	80.3–111.5	4.56–5.12	1.20–1.75
**Grass**	Sugarcane	-	1.22–1.59	19.35–20.96	10–40	55.60–57.40	23.90–24.50	24.35–26.30	0.31–1.25	257.3–290.5	15–18	6.20–8.2
	Corn stalks	-	0.50–1.50	10.00–20.00	33–39	38.33–40.31	25.21–32.22	7.32–21.45	0.21–0.38	33.40–34.80	4.10–4.50	1.90–2.30
	Rice straw	-	0.40–3.40	4.00–16.00	31–35	28.42–48.33	23.22–28.45	12.65–16.72	0.86–0.87	435–450	24.67–26.33	2.11–2.25

- **Water Retting (duration~7 to 14 days):** Plant stems or plant materials are soaked in water under a controlled condition and rely on natural microbial action to remove gummy materials. It produces fibers with uniform quality. High water requirements, foul odors, and high costs make this process less attractive.
- **Dew Retting (duration~14 to 21 days):** Plant stems or parts are harvested and left in a field for natural air decomposition of materials with pectinaceous properties. This is an easy method to extract gummy material, but the quality of the fibers will be inconsistent, and their properties will vary.
- **Enzymatic Retting (duration~12 to 24 h):** This process relies on specific enzymes such as pectinase and xylanase that act to remove pectinaceous substances under controlled conditions. This process produces low fiber strength material, which is a significant drawback.
- **Chemical Retting (duration~1 to 2 h):** This process requires the use of chemicals, such as sodium hydroxide, sodium benzoate, or hydrogen peroxide, for the removal of pectinaceous substances and is a more efficient method to produce long, clean fibers. The processing cost is one of the drawbacks, but it produces a fiber with a natural color and tensile strength.
- **Mechanical Processing or Mechanical Retting (duration~2 to 30 min):** This process is usually completed in only a few minutes. In a mechanical process, the fibers are decorticated, followed by cleaning, which produces many short fibers. Ball mills, hammer mills, or crushing rollers are used for decortication. The produced materials are sorted, screened, and size segregated for cleaning and use.

## 2. Agricultural Biomass Bio-Refining and Fiber Production

Millions of people are employed in natural fiber producing industries, especially those in developing countries where the industries consist of small-and large-scale operations. Fiber producing crops are cultivated throughout the world, but mostly in tropical and sub-tropical climates (mainly from developing countries). In recent years, the bioprocessing of cellulosic agricultural biomass for fiber production has gained momentum. Agricultural biomass, such as oil palm, bagasse, corn stalks, coir, bamboo, pineapple, banana, and rice husk are becoming useful. These are considered secondary sources of fiber. They are grouped as field residues and processed residues. Field residues are usually left unutilized in the field after harvest (e.g., banana), while the processed residues are biomass that is produced during the process of extracting the standard main product (e.g., bagasse). Table 3 shows the different agricultural fiber sources from diverse geographical locations that could serve as sources for fiber production, since agricultural residues comprise the surplus and low-cost materials to produce the fibers. Specific process integration and development are currently lacking. Based on the application of fibers, the process also needs to be tailored to calculate the cost of production, quantity, and quality of the fibers

### Extraction of Cellulosic Fibers from Agricultural Residues

The processing and extraction of cellulosic fibers from agricultural residues mainly aim to remove non-cellulosic content, including lignin, hemicellulose, and pectin (if present). Several factors govern the selection of extraction methods, but the chemical composition of agricultural residue is the key parameter. The most used methods for the extraction of cellulosic fibers from agricultural residues are:

- **Chemical method:** This method aims to remove non-cellulosic fiber materials. Sulfuric acid, hydrochloric acids, and hydrogen peroxide-based methods are widely reported to extract the fibers from agro-residues.
- **Mechanical method:** Mainly homogenization, ultrasonication, cryo-crushing, refining, grinding, micro fluidization, and electro spinning methods are developed and used. The steam explosion method has been developed, and it uses high pressure and temperatures to break the cell-wall fractions and separate the fibers.
- **Combined method:** The combination of chemical and mechanical methods is used more efficiently. This combination can produce high quantity fibers in a short retention time (e.g., high-pressure homogenization and sulfuric acid treatments).
- **Biological method:** The use of biological agents or enzymes to remove and extract cellulose are low-cost technologies. This method requires thorough optimization and process engineering for the different types of source materials used. Compared to chemical or mechanical methods, it is a time-consuming procedure, and the careful selection of biological agents is a key step to improve process efficiency.

In this section, we present a case study of the bio-refinery processing of residual biomass from banana plantations to produce fibers and value-added products.

## 3. Banana Plant and Residual Biomass: A Source for Natural Fibers and Value Products

**Figure 3:**
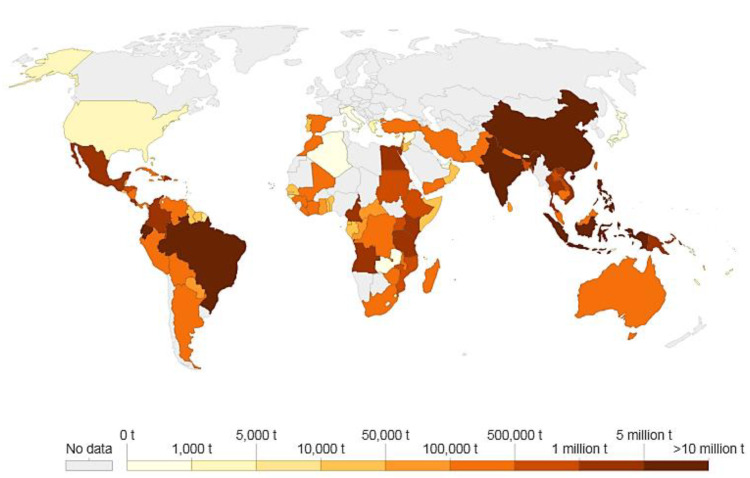
Global growth rates projected for banana production (2019–2024) (adapted from [21]).

Banana plants are grown in almost every country in the world, especially in tropical and subtropical climates (Figure 3). According to the Food and Agriculture Organization of the United Nations, global exports of bananas, excluding plantain, have reached an estimated quantity of 18.1 million tons per year. In 2017, a 6% increase in global exports was observed compared the numbers for 2016 [19]. According to the strong demand in the major markets, export volumes benefited from supply growth in key exporting countries, most notably those in Latin America. With the backing of enough supplies and a healthy demand, the net global import reached 17.4 million tons in 2017, an increase of 7% compared to 2016. The two largest net importers, the European Union and the United States of America, registered strong growth at rates of 7% and 5%, respectively. The average per capita consumption of bananas reached a peak of 13 kg in the United States of America and 12.6 kg in the European Union in 2017. [20] Bananas and plantains were grown in India all the way back to the Vedic times and are mentioned in Tamil literature dating back to 120 BC. The banana is one of the most important fruits in India, and it contributes to large productions and exports in the world markets. Canada has developed banana farms in Ontario that produce thousands of pounds of bananas. Although their banana production is comparatively less than that of other countries around the world, they are starting to cultivate the fruit using their own soil under greenhouse conditions. Banana cultivation generates significant biomass for handling or disposal, i.e., only 12% of plant parts are used, and the remaining 98% are discarded as waste. It is noted that the biomass produced from a banana plantation is 3- to 4-folds higher than that produced by other secondary fiber sources. By developing an integrated circular bioeconomy approach, every component of banana biomass could be recycled and potentially used [10].

Bananas are one of the earliest crops cultivated in the history of human agriculture. Many tropical and subtropical regions cultivate the land for banana plants. Land used for banana cultivation stretches from India to Papua New Guinea, as well as to the Southeast Asia region. Banana plant biomass and its by-products a remarkable source of priceless raw materials for other industries that recycle agricultural waste. For instance, the typical composition of banana residue is cellulose (50–60%), hemicellulose (25–30%), pectin (3–5%), lignin (12–18%), water-soluble materials (2–3%), fat and wax (3–5%), and ash (1–1.5%). Recycling this biomass helps to prevent the ultimate loss of a huge amount of untapped biomass and helps prevent environmental issues. Especially in banana farming, enormous quantities of biomass will mostly go to waste due to the non-availability of suitable technology for its commercial utilization. All types of banana species and waste from banana plantations could be used for fiber production. More research is needed to develop a sustainable workflow process for producing fibers and biorefinery concepts to maximize or re-purpose the value of residual biomass.

### 3.1. Classification and Taxonomy

Bananas are one of the largest herb groups in the world [4]. This plant can grow up to 7 m tall. It consists of spirally arranged oblong leaves, fleshy rhizomes (corm), and pseudo stems (leaf petioles). The female occupies the lower 5–15 rows, with deep purple waxy bracts and a long oval-shaped inflorescence and is supported by a stalk and the male flowers (upper rows). Wild types with seeded fruits are common, but the cultivated varieties are generally seedless, with virtually invisible dots of ovules at the center [12]. The term banana is frequently used to represent the dessert cultivar, while the cooking cultivar is generally referred to as a plantain. They belong to the family Musaceae and various species of the genus Musa have been cultivated since the beginning of human agricultural practices. They are utilized as a source of foods and ornaments [13].

### 3.2. Banana Fiber

Banana fibers, also known as musa fibers, are one of the world’s strongest natural fibers. Banana fiber is like natural bamboo fiber, but its spinnability, fineness, and tensile strength are said to be better. Banana fiber can be used to make several different textiles with different weights and thicknesses, based which part of the banana stem the fiber was extracted from. The thicker, sturdier fibers are taken from the banana trees’ outer sheaths, whereas the inner sheaths result in softer fibers. Banana fibers can be used to make ropes, mats, and woven fabrics, as well as handmade papers. Compared to various natural fibers, such as jute, coir, palm, etc., the banana fibers have a better tensile strength, i.e., 458 Mpa, with a 17.14 gigapascal modulus [22]. The banana fibers are biodegradable and include properties such as being fire-resistant, tear-resistant, and recyclable.

### 3.3. Extraction of Banana Fibers

**Figure 4:**
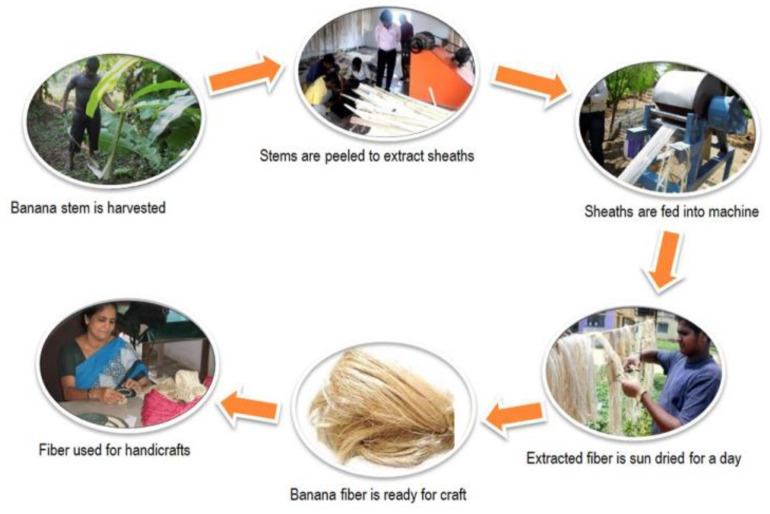
Mechanical separation of fibers from banana stems (adapted from [24]).

The extraction of fibers from residual plant biomass relies on chemical, mechanical, and biological methods. The chemical methods for extraction use NaOH, KMnO_4_, benzoyl chloride, stearic acid, etc., are not eco-friendly methods to use. The mechanical extraction methods are unable to remove the non-cellulose compounds; however, they are not replaceable for the processing and segregation of biomass. As shown in Figure 4, the typical process flow of extracting fibers from banana stems uses a simple mechanical process [23]. 

Traditionally, people processed the fibers using manual extraction methods, such as the splitting and scraping methods. They previously used knives and currently, the raspador machine is used for extracting fibers from banana pseudo stems. Other parts of the banana plant contain cellulose, which is extracted using biological methods for composite material preparations. The biological methods applied are solid-state fermentation and enzymatic methods, and they ultimately show promise for processing the biomass into fibers. There were excellent results that showed their ability to avoid fiber breakage. The most used enzymes are pectinase and xylanase [25]. Developing a process flow to effectively utilize the different biomass fractions are needed. The techno-economics and life cycle analysis of such process developments need to be established. 

### 3.4. Banana Fibers Used in Textile Productions

**Figure 5:**
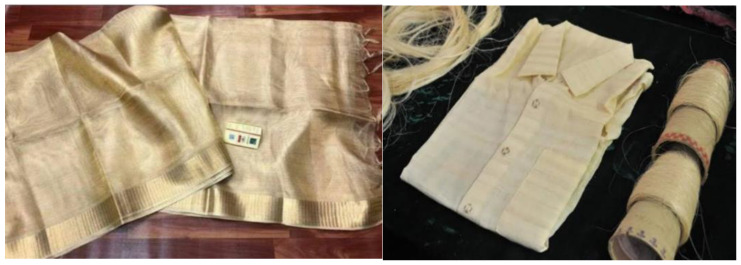
Traditional wares from banana silk [26].

Banana fabric is a beautiful, animal-free textile that mimics real silk, and acts as an excellent vegan alternative. The main material of the fiber comes from the stalk of the banana plant and is commonly referred to as banana silk (Figure 5). While it is certainly a unique idea, it is not recent. This textile was used by Japanese and Southeast Asian cultures as early as the 13th century. Banana fibers are a sustainable alternative to cotton and silk. The fibers have a natural sheen, and the inner strands of the stalk are extremely fine, allowing them to replicate the hand of silk. This textile is being used as an alternative to other fabrics as well. The quality of the fibers inside the stalk varies and the types of textiles produced can also vary. While the inner strands are smooth and fine, the outer strands are coarser. These fibers can be processed by hand, like bamboo, hemp, and linen. Some fibers are thick and coarse enough for basket weaving, and these can be used in handbags. The fibers have additional use in producing biodegradable packaging materials. 

### 3.5. Banana Biomass and Other Value Products

**Figure 6:**
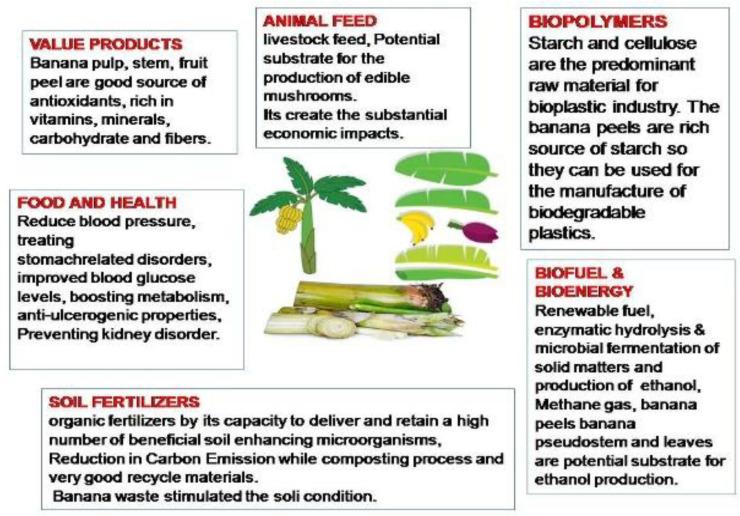
Various applications and uses of biomass from banana plantations.

The banana residual biomass is a good source to produce nutraceutical products, biofertilizers, biopolymers, nanomaterials, bioenergy, and biofuels (as shown in Figure 6). The effective utilization of the biomass, as well as standardized storage and handling procedures, are needed to ensure that the quality of the biomass remains stable prior to further processing. Moreover, the enormous varieties of the banana (Musaceae) family itself indicate an outstanding potential for exploration to obtain a standardized process flow to maximize the product recovery potential. For example, some bioactive constituents in a similar variety of banana plantations can vary significantly due to seasonal changes. These banana varieties may be identified by identical morphologies and cultivars; therefore, molecular identification is required to study or identify the banana varieties. The verification process can be expanded, further encompassing lesser varieties and intra- or interspecies.

## 4. Conclusions

Globally, there is a great deal of research that has recently been complete to find an alternative source for fiber production. Agricultural residues (e.g., banana residual biomass) are great secondary sources to be processed into natural fibers. The proper management of agricultural residues will help to significantly reduce the environmental problems caused by current disposal practices and support circular bioeconomy concepts that revolve around creating a framework of renewable resources, such as banana fibers, to be used for food management, health, land, and industrial systems using methods such as creating fibers from biomass waste. Bioeconomy economy is economy powered by nature. It is a new model of economy that uses the renewable product to reduce waste and replace the range of non-renewable waste. It is the idea of turning waste into a useful commodity for society [27]. With this circular bioeconomy concept for plant fibers, the biomass can benefit society by providing polymer production and other functionalities and properties from a waste product. The value of the end products and fibers will generate a potential income for banana farmers and the global industry. It also reduces the demand for fossil-derived polymer usage and is eco-friendly. However, there are challenges to be faced, such as the extent to which these biomass waste fibers can actually be utilized in society. Prospects for biomass waste utilization are promising, considering that the global production of this waste into fibers is continuing to rise every year, and additional methods to utilize this waste are currently being researched and developed. The banana plant was chosen as the key agricultural residue to focus on due to its massive global production volume and its excess biomass, which can be used for many different applications to reduce waste and contribute to sustainability in a circular bioeconomy. Currently, major climatic changes are caused by the development of industries and the mass production of synthetic materials. Therefore, we conclude that developing an integrated biorefinery option to produce fibers and other valuable products from the recycling of agricultural residues will be emphasized, and low-cost technologies should be developed to promote the circular bioeconomy. 

## Figures and Tables

**Table 1:** Type of important fibers (modified from [5,6]).

Fibers							
Natural Fibers					Man–Made Fibers		
Crop Fibers			Animal Fibers		Organic Fibers	Inorganic Fibers	
Seed fibers (Cotton)	Bast Fibers (Hemp, Kenaf, Ramie)	Hard Fibers (Sisal, Coco)	Wools Hairs (Wool, Angroa, Horsehair)	Silks	Natural Polymers (Viscose, Modal, Lyocell)	Synthetic Polymers (Polyester, Polyamide, Acrylic, Polypropylene)	(Carbon, Glass, Metal)

**Table 3:** Agricultural products as potential natural fiber resources [17,18].

Production (Megaton (MT) = 1 Million Tons)						
Countries	Banana	Sugarcane	Rice	Oil palm fruit	Wheat	Barley
Brazil	7.44	823.24	12.95	0.50	5.97	0.036
China	12.76	119.84	1.15	0.25	144.90	1.64
India	33.96	415.46	190.24	N/A	109.90	1.96
Indonesia	8.00	23.96	91.54	44.72	N/A	N/A
Malaysia	0.42	0.032	3.00	21.52	N/A	N/A
Philippines	6.77	27.26	21.02	0.14	N/A	N/A
Thailand	1.16	115.04	35.48	3.06	0.0015	0.031
Canada USA	N/A 0.0047	N/A 34.55	N/A 11.21	N/A N/A	35.02 56.54	9.24 3.67

Note: N/A—Not Available.

## Data Availability

Not applicable.
